# The Determinants of Psychological Well-Being Among Kidney Transplant Recipients in Kazakhstan: A Cross-Sectional Study

**DOI:** 10.3390/jcm14092894

**Published:** 2025-04-23

**Authors:** Aruzhan Asanova, Aidos Bolatov, Deniza Suleimenova, Gulnur Daniyarova, Aliya Sailybayeva, Sholpan Altynova, Yuriy Pya

**Affiliations:** 1 Department of Science, “University Medical Center” Corporate Fund, Astana 010000, Kazakhstan; asanova.aruzhan@umc.org.kz (A.A.); daniyarova.g@umc.org.kz (G.D.); s.aliya@umc.org.kz (A.S.); 2 School of Medicine, Shenzhen University, Shenzhen 518060, China; 3 School of Medicine, Astana Medical University, Astana 010000, Kazakhstan; 4 Clinical Academic Department of Pediatrics, “University Medical Center” Corporate Fund, Astana 010000, Kazakhstan; deniza.suleimenova@nu.edu.kz; 5 School of Medicine, Nazarbayev University, Astana 010000, Kazakhstan; 6 Department of Medical and Regulatory Affairs, “University Medical Center” Corporate Fund, Astana 010000, Kazakhstan; venera.altynova@umc.org.kz; 7 Clinical Academic Department of Cardiac Surgery, “University Medical Center” Corporate Fund, Astana 010000, Kazakhstan; yuriy.pya@umc.org.kz

**Keywords:** kidney transplantation, well-being, healthcare accessibility, post-transplant anxiety, patient satisfaction, Kazakhstan

## Abstract

**Background:** Kidney transplantation (KTx) significantly improves survival and quality of life in patients with end-stage renal disease. However, post-transplant well-being is influenced by multiple factors, including healthcare accessibility, satisfaction with medical care, and psychological health. This study aimed to assess the well-being of post-KTx patients in Kazakhstan and examine its associations with healthcare access, satisfaction, and anxiety. **Methods:** A cross-sectional study was conducted among 223 post-KTx patients in Kazakhstan. Participants were recruited through online surveys and telephone interviews. Only patients who had undergone transplantation within Kazakhstan were included. The WHO-5 Well-Being Index was used to measure well-being, and additional surveys assessed healthcare access, satisfaction with post-KTx care, and treatment-related anxiety. Multiple linear regression was performed to identify predictors of well-being. **Results:** The mean WHO-5 well-being score was 66.1 (SD = 24.6), indicating moderate well-being. Satisfaction with post-transplant information (β = 0.287, *p* = 0.015) and educational level (β = 0.172, *p* = 0.019) were significant positive predictors of well-being. In contrast, post-KTx anxiety (β = −0.154, *p* = 0.024) and difficulties in accessing medical care (β = −0.216, *p* = 0.014) negatively affected well-being. Patients residing in rural areas reported greater barriers to post-transplant care compared to those in urban settings (χ^2^ = 31.6, *p* = 0.002). **Conclusions:** Post-KTx well-being in Kazakhstan is influenced by educational level, access to healthcare, satisfaction with medical information, and anxiety levels. Targeted interventions to improve access to post-transplant care, enhance patient education, and address psychological distress may help improve outcomes for post-KTx patients.

## 1. Introduction

Chronic kidney disease (CKD) is a progressive condition that leads to a gradual decline in kidney function, ultimately resulting in end-stage renal disease (ESRD), a stage at which patients require either dialysis or a kidney transplant (KTx). The global incidence of CKD has been steadily rising, contributing to an increasing number of individuals in need of KTx. In Kazakhstan, a retrospective analysis spanning from 2014 to 2020 revealed a notable increase in the prevalence of CKD from 10,346 to 38,287 cases per million population (pmp). The mortality rate associated with CKD has also increased, and projections indicate further growth in both prevalence and mortality rates in the coming years [1].

KTx remains the preferred treatment for individuals with ESRD, offering significant improvements in both health-related quality of life (HRQoL) and life expectancy [2]. Furthermore, KTx is considered a more cost-effective option than prolonged dialysis therapy [3,4]. Since 2012, the number of KTxs performed in Kazakhstan has steadily increased, with over 150 operations conducted annually. As of January 2025, a total of 2099 KTxs have been carried out across the country, including 200 from deceased donors, according to the Republican Center for Coordination of Transplantation and High-Tech Medical Services [5]. Despite these advancements, Kazakhstan’s healthcare system still faces challenges with the management of post-KTx patient outcomes, making it crucial to understand the factors influencing the post-transplant life quality of kidney recipients.

Studies indicate that health-related quality of life (HRQoL) improves significantly after KTx compared to before the procedure and is superior to that of patients undergoing dialysis [6]. However, the HRQoL post-KTx is still considerably lower than that of the general population [6]. The well-being of post-KTx patients is shaped by a variety of clinical and nonclinical determinants, including physical health, healthcare access, satisfaction with medical care, psychological and emotional health, and others. Research indicates that over time, HRQoL can decline due to factors such as the progression of underlying comorbidities and the development of new health issues after transplantation [6]. Moreover, the long-term use of immunosuppressive drugs after transplantation presents physical, psychological, and social challenges due to the side effects associated with these drugs.

Post-traumatic stress disorder is another significant concern affecting the life quality of kidney recipients. The transplantation process itself can be traumatic, with patients often experiencing feelings of panic, anxiety, and emotional distress. Such traumatic experiences can lead to post-traumatic stress, which, in turn, may contribute to medication non-adherence and other negative outcomes in transplant recipients, significantly reducing their life quality [6]. Another significant factor is fatigue, which affects nearly half of kidney transplant recipients and is strongly associated with a poorer quality of life, impairing overall functioning across multiple health domains [7]. Uncertainty and anxiety about potential rejection and graft loss is one of the factors negatively affecting post-KTx patients [8]. Research on return to work among transplant recipients has revealed that psychological support and managing psychological fragility during the post-transplant period are important factors that predict the likelihood of returning to work [9]. This highlights the importance of addressing not only the physical health of transplant recipients but also their psychological well-being in order to optimize overall outcomes.

Environmental factors play an equally important role in the recovery and return of patients after KTx to their regular life. Studies demonstrate that access to medical care, the availability of comprehensive health information about the post-transplant period, and the management of chronic illnesses can also greatly influence HRQoL [10,11]. Meanwhile, a shortage of healthcare professionals or an insufficient or absent healthcare infrastructure can hinder proper post-transplant care and follow-up. Furthermore, patients’ education level is another predictor of post-transplant quality of life. Higher education levels are often associated with better adherence to treatment regimen and maintaining financial stability after transplantation, which in turn contribute to an improved quality of life [9]. Therefore, a holistic approach to post-transplant care, one that incorporates both physical and mental health considerations and environmental factors, is crucial for achieving optimal outcomes for kidney transplant recipients.

This study aims to evaluate the well-being of post-KTx patients in Kazakhstan through a cross-sectional approach, identifying key factors that influence their quality of life, such as healthcare access, satisfaction with care, and anxiety levels. By exploring the relationships between these factors and patient well-being, this study offers valuable insights that can inform targeted interventions to improve the overall post-transplant experience.

## 2. Materials and Methods

### 2.1. Study Design and Participants

This cross-sectional study was conducted among post-kidney transplant (KTx) patients in Kazakhstan. Patients were recruited through online and telephone-based surveys. The online survey link was distributed via patient support group chats, while additional participants were contacted by phone. Only patients who had undergone kidney transplantation within Kazakhstan were included in the analysis. Responses from 10 patients who had undergone KTx abroad and 1 patient with a second-time kidney transplantation were excluded, resulting in a final sample of 223 participants.

### 2.2. Data Collection

Participants completed a structured questionnaire that included socio-demographic characteristics (age, gender, residence, educational level, employment, and family status), clinical variables (comorbidities, duration of chronic kidney disease before KTx, and time since transplantation), and measures related to healthcare accessibility, satisfaction with medical care, and psychological well-being.

### 2.3. Measures

#### 2.3.1. Well-Being Assessment

Well-being was assessed using the WHO-5 Well-Being Index, a validated tool for measuring subjective psychological well-being. The index consists of five statements, each rated on a 5-point Likert scale (0 = at no time, 5 = all of the time). The raw score ranges from 0 to 25, where 0 represents the worst possible mental well-being and 25 represents the best possible mental well-being. To calculate a percentage score (ranging from 0 to 100), the raw score was multiplied by four, with 0 representing the worst possible well-being and 100 representing the best possible well-being.

#### 2.3.2. Satisfaction with Post-Transplant Medical Care

Patient satisfaction with post-KTx medical care was assessed across four key areas: (1) communication with medical staff; (2) the amount and quality of information received about life after transplantation; (3) the level of post-KTx care (including condition monitoring, counseling, and post-KTx check-ups); and (4) the quality of post-KTx care at the registered primary healthcare facilities. Each aspect was rated on a 5-point Likert-type scale, ranging from 1 (very dissatisfied) to 5 (very satisfied).

#### 2.3.3. Access to Healthcare and Medications

Participants were asked about their frequency of difficulties accessing healthcare services or medications post-transplantation using a 5-point Likert-type scale: never, rarely, sometimes, often, or always.

#### 2.3.4. Availability of Post-Transplant Follow-Up Care

Perceived availability of follow-up care was assessed, with responses categorized into three levels: limited availability (unavailable, very unavailable); moderate availability; sufficient availability (available, very available)

#### 2.3.5. Post-Transplant Health and Treatment-Related Anxiety

Patients’ concerns regarding post-KTx health and treatment were assessed through seven questions, each rated on a 5-point scale (never, rarely, sometimes, often, always) based on Rhu et al.’s (2019) study [12]. These included concerns about the following: (1) the risk of infection due to immunosuppressants; (2) kidney function maintenance; (3) poor laboratory test results; (4) long-term health stability; (5) the financial burden of treatment; (6) emotional well-being (depression or low mood); and (7) the side effects of immunosuppressive medications. A higher score indicated greater anxiety related to post-KTx health and treatment.

### 2.4. Statistical Analysis

Descriptive statistics were used to summarize the demographic and clinical characteristics of the participants. Categorical variables were presented as frequencies and percentages, while continuous variables were reported as means and standard deviations (SDs). Associations between residence type and healthcare accessibility were examined using chi-square (χ^2^) tests. The Mann–Whitney U test was used to compare non-normally distributed continuous variables, including well-being and anxiety levels, between independent age groups. A multiple linear regression analysis was conducted to identify predictors of well-being, with variables including demographic factors (age, gender, education, residence, employment, family status), health-related variables (comorbidities, CKD duration), healthcare accessibility, satisfaction with care, and post-KTx anxiety. In addition, mediation analyses were conducted to assess the indirect effects of key variables on well-being. Model 1 tested the indirect effect of place of residence on well-being via difficulties in accessing healthcare services. Model 2 examined the indirect effect of educational level on well-being through difficulties with access to healthcare and medications. Statistical significance was set at *p* < 0.05. All analyses were performed using Jamovi software (version 2.6.17).

### 2.5. Ethical Considerations

This study was conducted in accordance with the principles outlined in the Declaration of Helsinki. Ethical approval was obtained from the Local Bioethics Commission of the “University Medical Center” Corporate Fund (Protocol No. 3 dated 14 July 2023) prior to data collection. All participants were provided with detailed information about the purpose, procedures, and voluntary nature of the study. Informed consent was obtained from all participants electronically (via online surveys) or verbally (for telephone interviews) before participation. Anonymity and confidentiality were strictly maintained throughout the study. No personal identifiers were collected, and all responses were securely stored and accessible only by the research team. Participation did not pose any physical or psychological risk to respondents, and individuals could withdraw from the study at any time without consequence.

## 3. Results

This study included 223 post-KTx patients, with an average age of 38.7 (SD = 10.4) years, ranging from 18 to 64 years. In terms of age distribution, 52.9% were aged 18–39 years, and 47.1% were aged 40–64 years. The average time since kidney transplantation (KTx) was 57.0 months (SD = 47.0). The sample comprised 98 males (43.9%) and 125 females (56.1%). Table 1 presents the main socio-demographic characteristics of the study group.

Regarding residential distribution, 38.6% of participants resided in national-level cities (Astana, Almaty, and Shymkent), 33.6% in regional-level cities, 14.8% in rural areas, and 13.0% in small towns. Educational levels varied, with the majority of participants having an undergraduate degree (58.3%), followed by special education (26.0%), high school (9.9%), middle school (4.9%), and postgraduate education (0.9%).

In terms of employment, 36.8% were employed, 30.9% were on disability benefits, 11.7% were self-employed, 8.5% were students, 8.1% were unemployed, and 4.0% were pensioners. The majority of participants were married (60.5%), while 29.6% were single, 7.6% divorced, and 2.2% widowed. Additionally, 64.6% had children, while 35.4% did not.

The prevalence of comorbidities was high, with 62.8% of patients reporting at least one comorbidity, while 37.2% had none. Regarding the duration of kidney disease before transplantation, 36.3% had chronic kidney disease for more than 5 years, 33.2% for 1–3 years, 20.6% for less than 1 year, and 9.9% for 4–5 years.

### 3.1. Disparities in Access to Post-Transplant Healthcare and Medications

Participants reported varying levels of difficulty in accessing healthcare services and medications after transplantation. Specifically, 10.8% of patients never experienced access issues, while 25.6% encountered them rarely. However, a significant proportion reported difficulties, with 37.7% experiencing access issues sometimes, 7.2% often, and 18.8% always. These findings suggest that despite some degree of accessibility, a substantial number of post-KTx patients face ongoing challenges in obtaining necessary medical care and medications.

Further analysis of access issues based on residence revealed significant disparities (χ^2^ = 25.4, *p* = 0.013). Patients living in rural areas faced the highest barriers, with 42.4% reporting persistent difficulties (always), compared to 27.6% in small towns, 14.7% in regional-level cities, and 10.5% in national-level cities. Conversely, only 15.2% of rural residents reported never having access issues, compared to 9.3% in national-level cities, 10.7% in regional-level cities, and 10.3% in small towns. These findings highlight that patients in rural and small urban settings are disproportionately affected by limited access to medications.

### 3.2. Post-Transplant Follow-Up Care: Patterns of Availability

The perceived availability of post-transplant follow-up care varied among participants. Overall, 23.3% of patients rated follow-up care as very difficult to access, while 13.5% found it somewhat difficult. A moderate level of availability was reported by 33.2%, whereas 18.8% considered follow-up care accessible, and 11.2% rated it as very accessible. These findings indicate that while a portion of the population experiences adequate access to post-transplant care, a significant number of patients still face challenges.

A comparison based on residence revealed notable disparities in accessibility (χ^2^ = 31.6, *p* = 0.002). For analysis, responses were grouped into three categories: limited availability (very unavailable and unavailable), moderate availability (middle), and sufficient availability (available and very available). Patients residing in rural areas were the most likely to report limited availability (60.6%), followed by those in small towns (51.7%). In contrast, regional-level city (30.7%) and national-level city (24.5%) residents reported fewer difficulties in accessing follow-up care. The highest proportion of respondents perceiving care as moderately available was in regional-level cities (41.3%) and national-level cities (33.7%), compared to rural areas (15.2%). In terms of sufficient availability, national-level city residents had the highest proportion (41.9%), followed by regional-level cities (24.0%). In contrast, only 24.3% of rural residents and 17.2% of small-town residents perceived care as sufficiently available.

These findings highlight significant disparities in post-transplant care accessibility based on geographic location, with rural and small-town residents facing the greatest challenges. The results underscore the need for targeted healthcare policies to improve access to follow-up care, particularly for patients outside major urban centers.

### 3.3. Reported Satisfaction with Post-Transplant Support and Monitoring

The highest satisfaction was observed in communication with medical staff, where 50.7% of respondents were very satisfied and 30.9% were satisfied, while only 0.4% were very dissatisfied and 4.9% were dissatisfied (Table 2).

Regarding the amount and quality of information received about life after transplantation, 40.8% of participants were very satisfied, and 33.6% were satisfied, whereas 1.3% were very dissatisfied and 8.1% were dissatisfied.

For post-transplant medical care quality, including condition monitoring, counseling, and check-ups, 39.5% of patients were very satisfied, 29.1% were satisfied, and 19.7% were partially satisfied. However, 9.0% expressed dissatisfaction, and 2.7% were very dissatisfied.

Satisfaction with the quality of post-transplant medical care at the registered primary healthcare facility was lower compared to other aspects. Only 29.6% of patients were very satisfied, while 28.3% were satisfied. A notable 11.2% were dissatisfied, and 7.2% were very dissatisfied, indicating room for improvement in primary healthcare settings.

### 3.4. Health- and Treatment-Related Anxiety Among Transplant Recipients

Patients reported varying levels of health- and treatment-related anxiety post-transplantation, with an overall mean anxiety score of 3.65 (SD = 1.00), ranging from 1 to 5. The most common concern was the functionality of the transplanted kidney, with 46.2% of respondents always worrying about how well it will be maintained, and another 26.0% expressing concern sometimes. Similarly, 41.7% always worried about poor laboratory test results, and 46.2% were consistently concerned about their long-term health stability (Table 3).

Financial concerns related to post-transplant treatment were also prevalent, with 46.6% of participants always worried about associated costs, and 21.5% sometimes experiencing financial anxiety.

Concerns about the risk of infection due to immunosuppressive therapy were frequently reported, with 40.4% always feeling anxious and 27.8% sometimes experiencing concern. Additionally, 34.1% of patients always worried about potential kidney complications due to immunosuppressive medication.

Mental health issues, such as post-transplant depression or low mood, were reported by 23.3% of patients always and 8.5% often, while 22.9% rarely experienced depressive symptoms.

To examine age-related differences in psychological burden, post-KTx health and treatment-related anxiety levels were compared between two age groups: 18–39 years and 40–64 years. The Mann–Whitney U test was used to assess differences in total anxiety scores, and the chi-square test was applied to individual anxiety components. No statistically significant differences were observed between the two age groups in either the total anxiety levels or any individual components (all *p* > 0.05). These findings suggest that post-transplant health and treatment-related anxiety is experienced similarly across age groups, highlighting the broad relevance of psychological support interventions regardless of age.

### 3.5. Psychological Well-Being

The general well-being of post-KTx patients, as measured by the WHO-5 Well-Being Index, was moderate, with a mean score of 66.1 (SD = 24.6) in the range of 0 to 100. In our sample of 223 post-KTx patients, approximately 74% scored in the upper three quartiles of the WHO-5 Well-Being Index (Quartiles 2–4), 51% fell into the middle two quartiles (Quartiles 2–3), and about 23% scored in the highest quartile (Quartile 4). A multiple linear regression analysis was conducted to identify factors influencing well-being among post-KTx patients. The model was statistically significant (R^2^ = 0.242, F = 2.63, *p* < 0.001), explaining 24.2% of the variance in well-being scores (Table 4).

Among the demographic factors, educational level was a significant predictor of well-being (β = 0.171, *p* = 0.019), indicating that higher education was associated with better well-being outcomes. Divorced individuals reported significantly lower well-being scores compared to single participants (β = −0.676, *p* = 0.031). Having children, gender, age, and place of residence did not have statistically significant associations with well-being. However, mediation analysis revealed a significant indirect effect of place of residence on WHO-5 well-being scores through difficulties with access to medical care (Table 5). Specifically, residence in small towns or rural areas was associated with more pronounced issues in accessing healthcare services (*p* < 0.001), which was in turn strongly linked to lower well-being (*p* < 0.001). The direct and total effects of residence on well-being were not statistically significant (*p* = 0.499). These findings suggest that although residence does not independently predict well-being, it may influence post-transplant outcomes through access-related barriers to medical care, highlighting the importance of healthcare accessibility as a mediator of geographic disparities in quality of life.

Health-related factors did not influence well-being. The presence of comorbidities was associated with lower well-being (β = −0.240, *p* = 0.085), though the effect was not statistically significant at the 0.05 level. Longer chronic kidney disease duration before transplantation also was not significantly associated with well-being (*p* = 0.346).

Regarding access to healthcare, difficulties in obtaining medical care were significantly associated with lower well-being scores (β = −0.216, *p* = 0.014), suggesting that patients who faced challenges in accessing post-transplant medical services experienced worse well-being. However, issues with access to medications were not significantly associated with well-being (*p* = 0.884).

Patient satisfaction with post-transplant care played a role in well-being. Specifically, higher satisfaction with the quality and quantity of post-transplant information was a strong positive predictor of well-being (β = 0.287, *p* = 0.015). Finally, post-transplant anxiety had a significant negative effect on well-being (β = −0.154, *p* = 0.024), indicating that patients with higher levels of health-related anxiety experienced poorer overall well-being.

Difficulties with access to healthcare and medications were not significantly associated with well-being in the standard regression model (*p* = 0.884). However, the mediation analysis revealed a significant indirect effect of educational level on well-being through this variable (Table 6). Specifically, educational level was indirectly associated with WHO-5 well-being scores via issues with access to medical care (*p* = 0.037). Lower educational attainment was linked to greater reported difficulties in accessing healthcare and medications (*p* = 0.016), and these difficulties, in turn, were negatively associated with well-being (*p* < 0.001). The direct effect of educational level on well-being remained significant (*p* = 0.003), as did the total effect (*p* < 0.001). These findings indicate that while access difficulties alone may not predict well-being directly, they serve as a significant pathway through which education influences well-being. At the same time, no significant interaction effects were observed between educational level and occupational status or satisfaction with information in predicting well-being.

Overall, educational level, satisfaction with transplant-related information, and access to medical care were key positive contributors to well-being, while divorce and post-transplant anxiety negatively impacted well-being. These findings highlight the importance of improving healthcare accessibility, providing comprehensive post-transplant information, and addressing psychological distress to enhance well-being outcomes in post-KTx patients.

## 4. Discussion

This study aimed to investigate subjective well-being among post-kidney transplant (KTx) patients in Kazakhstan and to determine how healthcare accessibility, satisfaction with care, and post-transplant anxiety affect well-being. Overall, participants’ well-being, as measured by the WHO-5 Index, was moderate (mean score: 66.1), indicating that while many patients maintain satisfactory psychosocial health after transplantation, there is considerable room for improvement. Educational level and satisfaction with transplant-related information emerged as strong positive predictors of well-being, whereas the presence of healthcare accessibility challenges and elevated post-KTx anxiety were negatively associated with it. Furthermore, patients in rural areas reported notably greater barriers in accessing follow-up care and essential medications compared to their urban counterparts, underscoring the influence of geographic disparities on post-transplant outcomes.

The moderate WHO-5 well-being score underscores that although kidney transplantation can significantly enhance survival and overall quality of life, post-transplant patients are still vulnerable to psychosocial stressors. When comparing these findings to an Ethiopian study of living kidney donors and recipients, certain similarities and differences emerge. In that study, 83% of the transplant recipients fell into the upper three quartiles of psychological well-being, a slightly higher proportion than the 74% in our sample. Meanwhile, about half of the Ethiopian sample (50%) reported scores in the middle two quartiles, closely aligning with the 51% observed among our participants [13].

### 4.1. The Role of Education and Knowledge

The positive association between higher educational attainment and better well-being is consistent with the broader literature suggesting that individuals with more formal education may have greater capacity to navigate healthcare systems and make informed decisions about their post-transplant care [14,15,16]. Thus, a recent analysis of 30,999 adult heart transplant patients (1999–2018) found that having less than a high school education was associated with a 17% higher risk of post-transplant death, even after adjusting for factors like age and comorbidities. Patients who had education beyond high school not only lived longer on average but also had fewer organ rejection episodes and better follow-up compliance in their care [17]. Moreover, education can enhance coping skills and confidence in disease self-management. A comprehensive study on health-related quality of life (HRQoL) after various solid organ transplants identified “limited education” as one of the factors that negatively influenced post-transplant quality of life in a multivariate analysis [18]. The relationship between education and transplant outcomes is also recognized in broader reviews. The review by Green & Cavanaugh (2015) [19] summarized that patients with lower education levels have higher risks of progression to end-stage renal disease, more complications on dialysis, worse post-transplant outcomes, and higher mortality. The mechanisms behind these disparities are complex and interrelated—involving differences in health knowledge, health behaviors (like medication adherence and diet), financial and insurance factors, and psychosocial support networks [19]. Similarly, feeling well informed about life after transplantation, reflected in higher satisfaction with transplant-related information, likely empowers patients to engage more confidently in self-management, adhere to immunosuppressive regimens, and seek prompt medical attention when needed. In line with the literature, our findings demonstrate that higher satisfaction with the quality and quantity of post-transplant information was a strong positive predictor of well-being (β = 0.287, *p* = 0.015). Difficulties with access to healthcare and medications were not significantly associated with well-being in the standard regression model (*p* = 0.884). However, mediation analysis revealed a significant indirect effect of educational level on well-being through this variable (*p* = 0.037). Specifically, educational level was indirectly associated with WHO-5 well-being scores via issues with access to medical care. These findings suggest that while access difficulties alone may not directly predict well-being, they serve as an important pathway through which education influences post-transplant outcomes. At the same time, no significant interaction effects were observed between educational level and either occupational status or satisfaction with information in predicting well-being.

### 4.2. The Role of Residence and Access to Healthcare

Evidence indicates potential urban–rural gaps in post-transplant quality of life. A cross-sectional study from Nepal found that kidney transplant recipients in urban areas reported significantly better HRQoL than those in rural areas. In this study, urban residence was associated with higher QoL scores in transplant patients, along with higher socioeconomic status and education [20]. Rural transplant recipients likely face challenges such as travel to clinics, financial strain, and reduced local healthcare support, which can detract from their perceived well-being. Conversely, some research suggests that transplant can mitigate pre-existing QoL disparities. A pediatric kidney transplant study in the U.S. found that children living >30 miles from the transplant center (mostly rural) had lower quality of life while on dialysis but experienced larger post-transplant improvements. In fact, transplantation yielded even greater HRQoL gains for patients from remote/rural regions, effectively narrowing the gap in well-being by reducing the burden of frequent medical visits [21]. This underscores that the post-transplant quality of life for rural patients is highly sensitive to healthcare access demands.

Although place of residence was not directly associated with well-being in our regression model, further analysis revealed that it plays a critical indirect role in shaping post-transplant outcomes through its effect on healthcare accessibility. Patients residing in rural areas and small towns reported significantly greater challenges in accessing both medications and follow-up care compared to those living in regional- or national-level cities. Specifically, 42.4% of rural residents reported persistent difficulties in accessing medical services, and 60.6% reported limited availability of follow-up care, substantially higher than their urban counterparts. Mediation analysis confirmed that these access barriers significantly contributed to lower well-being scores (*p* = 0.002). While residence itself did not independently predict well-being (*p* = 0.499), its influence was exerted through increased difficulty in accessing care (indirect effect: *p* < 0.001), which in turn was strongly associated with reduced well-being (*p* < 0.001). These findings underscore that geographic disparities, particularly those affecting healthcare access, can translate into inequities in post-transplant quality of life. Interventions aiming to improve transplant outcomes in Kazakhstan must therefore prioritize reducing structural barriers to care in rural and underserved areas. Moreover, a lack of accessible healthcare services negatively affected patients’ well-being. This finding points to systemic challenges in the healthcare infrastructure, particularly in rural settings, that can hamper ongoing post-transplant care.

In Kazakhstan, specialized nephrology services are primarily concentrated in regional and national medical centers. In rural areas and small towns, patients often lack direct access to nephrologists and are typically followed-up by general practitioners or internists in local outpatient settings. When complications arise, they may be referred to regional centers for hospitalization, receive scheduled in-person consultations at transplant centers, or utilize telemedicine consultations with specialists. These systemic limitations in specialist care likely exacerbate the challenges reported by rural and small-town patients and may help explain the significant indirect association between residence and well-being observed in our mediation analysis. This highlights the need for decentralized, community-integrated nephrology support and digital care pathways to ensure equitable long-term follow-up.

Crucially, research suggests that healthcare access barriers associated with rural residence mediate many of the disparities in outcomes and well-being. Distance is a key factor: rural Americans often live hours from transplant centers, and longer travel distances have been linked to worse pre- and post-transplant metrics. For example, liver disease patients living >150 miles from a transplant center had significantly higher mortality (including while waiting) than those living near a center [22]. Travel hardship can also continue after transplant, as rural recipients face lengthy trips for routine follow-ups, laboratory monitoring, or managing complications. This burden is reflected in patient surveys and provider reports: a scoping review identified “the need to travel or relocate to access required medical testing and transplantation facilities” as the predominant barrier for rural transplant candidates. The same review noted substantial physical and psychosocial impacts stemming from these access difficulties; patients from remote areas reported more stress, financial strain, and disruption to family life in the course of seeking a transplant [23]. Such factors can directly undermine post-transplant well-being, for instance by causing anxiety, delaying follow-up care, or reducing adherence to medications and appointments.

Apart from geography itself, associated social determinants of health play a mediating role. Rural communities often have higher poverty rates and fewer healthcare resources, which contribute to disparities. A recent review pointed out that poverty and related social risk factors likely “mediate rural-urban disparities” in transplant outcomes. Lower density of specialists in rural areas (e.g., fewer hepatologists or nephrologists to refer patients) and gaps in insurance coverage (especially in non-expansion rural states) can translate into delayed care and poorer health status at transplant [22]. These disadvantages can carry into the post-transplant phase as well, affecting one’s ability to access rehabilitation, pharmacy services, or mental health care. Indeed, analyses of community risk indices show that patients from more socioeconomically distressed areas have modestly worse long-term transplant survival, likely reflecting ongoing barriers in care continuity and support [24]. Thus, awareness of these residence-driven inequities is vital in transplant medicine; it calls for targeted support to ensure that transplant recipients, no matter where they live, can achieve the highest possible well-being and quality of life after their life-saving surgery. The significant rural–urban gap in follow-up care availability and medication access highlights how geography can amplify vulnerabilities among transplant recipients. The limited availability of transplant centers, specialized medical staff, and consistent medication supplies in rural regions can create additional logistical and financial burdens. Consequently, these barriers can translate into poorer clinical follow-up and higher distress.

### 4.3. The Role of Post-Transplant Health and Treatment-Related Anxiety

Transplant recipients often face health-related anxieties specific to their condition and lifelong medical regimen. Key sources of post-transplant anxiety include the following:

− Fear of Graft Failure or Rejection: Many patients live with a persistent worry that their transplanted organ could fail. In a heart transplant cohort, 28% perceived graft rejection as a serious threat, and those with poorer well-being reported more intrusive anxiety about rejection. This fear of graft loss can be an ongoing stressor strongly tied to psychological well-being [25].
− Concerns Over Medical Results and Complications: Routine follow-ups, lab tests, and biopsies can trigger anxiety as patients await results. Uncertainty about health status often leads to preoccupation with possible negative outcomes. Transplant recipients with chronic medical conditions frequently worry about complications or recurrence of illness, which is considered a normal response up to a point. However, if this worry becomes excessive, it may cross into health anxiety that impairs daily functioning [26].
− Risk of Infections and Other Illnesses: Because immunosuppressive therapy elevates infection risk, patients may experience heightened anxiety about exposure to infections (e.g., seasonal illness or COVID-19) and their ability to fight them [27]. Such fears were magnified during the COVID-19 pandemic, when transplant recipients reported substantial psychological distress and worry about contracting infections, contributing to overall anxiety levels and lower quality of life [28].
− Medication Side Effects and Regimen Demands: Lifelong immunosuppressant use comes with side effects and strict adherence requirements. Patients may worry about the long-term effects of these medications on their health or fear missing doses. The need for continuous medication monitoring and potential side effects can be a source of chronic stress [27].
− Financial Burden: The cost of transplant care and medications can cause significant anxiety. Nearly one in four liver transplant recipients in a recent multicenter study reported high financial burden (spending ≥10% of income on medical costs), and this financial stress was linked to worse daily functioning and even delayed or skipped medical care. Those experiencing high financial strain also reported markedly lower health-related quality of life compared to patients with less financial burden [29].

In the current study, health and treatment-related anxiety emerged as a significant factor negatively impacting well-being among post-KTx patients in our study. With a mean anxiety score of 3.65 (on a 1–5 scale), participants frequently expressed concerns about their post-transplant health. The most prevalent sources of anxiety included fear of graft failure (46.2% always worried), long-term health instability (46.2%), and poor laboratory results (41.7%). Financial concerns were also prominent, with nearly half of the participants (46.6%) consistently worried about the cost of ongoing treatment, highlighting the psychological burden associated with the economic demands of lifelong care. Anxiety related to immunosuppressive therapy was widespread, with many patients expressing persistent fear of infection and medication side effects. Emotional distress was also common, with 23.3% of respondents always experiencing symptoms of depression or low mood. Consistent with these findings, post-transplant anxiety showed a significant negative association with well-being (β = −0.154, *p* = 0.024), indicating that individuals experiencing higher anxiety had lower psychological well-being.

These results align with previous research highlighting the psychological burden faced by transplant recipients. Studies have consistently shown that anxiety is prevalent among post-transplant populations, often stemming from fears of graft failure, medication adherence, and future health uncertainty. For instance, Rodrigue et al. (2010) [30] found that anxiety symptoms were common among kidney transplant recipients and were associated with poorer HRQoL. A Chinese survey during COVID-19 found that organ transplant recipients with clinically significant anxiety, depression, insomnia, or PTSD had poorer quality of life across all domains compared to those without such symptoms [28]. Similarly, heart transplant patients who exhibited high health anxiety (health-related hypochondriasis) were more likely to report impaired psychological well-being and distress. In some studies, over one-third of heart recipients showed “clinically significant health anxiety”, a rate double that of healthy controls [26]. This heightened anxiety correlates with feeling less in control of one’s health and more helpless in the face of potential problems, which erodes mental quality of life. Importantly, excessive anxiety can be psychologically disabling; when worry becomes overwhelming, it significantly impairs patients’ emotional quality of life as they cope with constant concerns [26]. On the other hand, successful transplantation can improve many patients’ mental health over time; one prospective study noted significant reductions in anxiety and depression scores by one year after liver transplant, corresponding with improved quality of life [31]. Nonetheless, a considerable proportion continue to struggle with anxiety long-term, indicating that improvements are not universal and mental health support remains crucial.

Anxiety is now recognized as a significant factor affecting the long-term well-being of transplant recipients, cutting across transplant medicine, psychiatry, behavioral science, and public health domains. From the transplant medicine perspective, improving survival and graft function is not enough—attention must also be paid to patients’ mental health and anxiety levels, as these can influence adherence and even survival [32,33]. Psychiatry and behavioral science experts emphasize the need for routine screening and treatment of anxiety disorders in transplant populations, given the high prevalence of post-transplant anxiety and its detrimental impact on quality of life [26,28]. Interventions like cognitive behavioral therapy, stress management, and psychoeducation about normal vs. pathological health anxieties can help patients cope with fears of rejection, infection, and other concerns. Importantly, not all anxiety is maladaptive—a normal degree of health anxiety can motivate patients to attend follow-ups and adhere to care. But when anxiety becomes excessive or chronic, it requires intervention to prevent impairment in daily functioning and well-being [26].

Public health and psychosocial researchers also highlight financial toxicity and social support as crucial components of well-being. Transplant centers are encouraged to screen for financial stress and connect patients with resources, since financial anxiety can compound psychological distress and lead patients to forego necessary care [29]. Social workers, psychologists, and support groups can be integrated into transplant aftercare to address these broader determinants of mental health. In summary, anxiety, particularly centered on health and graft-related issues, plays a meaningful role in shaping transplant recipients’ mental health outcomes and overall life satisfaction after surgery. A growing body of interdisciplinary research shows that managing anxiety is key to optimizing post-transplant well-being, alongside medical management of the transplant itself. Ensuring patients feel psychologically supported may improve not only their quality of life but potentially their transplant success in the long run [25,27].

### 4.4. Clinical and Policy Implications

The findings of this study have several important clinical and policy implications for improving the quality of life and psychological well-being of kidney transplant (KTx) recipients in Kazakhstan.

1. Strengthening Post-Transplant Care in Rural Areas

Geographic disparities in access to follow-up care and medications were strongly linked to reduced well-being among rural and small-town residents. Although place of residence was not a direct predictor of well-being, it had a significant indirect effect through healthcare accessibility barriers. These findings emphasize the need for targeted policies to expand post-transplant services beyond major cities. This could include establishing satellite clinics, enhancing transportation support, and integrating telemedicine into transplant follow-up, particularly for rural regions.

2. Integrating Mental Health Support into Transplant Aftercare

A substantial proportion of patients reported high levels of health and treatment-related anxiety, especially concerns about graft failure, financial burden, infection risk, and long-term health stability. Anxiety was found to be a significant negative predictor of well-being. This highlights the necessity for routine psychological screening and the integration of mental health services, such as counseling, cognitive behavioral therapy, and peer support, into standard post-KTx care. Addressing mental health needs is not only essential for improving quality of life but may also enhance treatment adherence and clinical outcomes.

3. Enhancing Patient Education and Health Literacy

Higher satisfaction with transplant-related information and higher educational attainment were both associated with better well-being. Educational interventions—delivered through personalized counseling, printed materials, or digital platforms—should focus on improving patients’ understanding of graft care, medication management, and the long-term implications of immunosuppression. Tailoring education efforts to patients with lower literacy levels or educational backgrounds may help reduce disparities in self-management capacity and health outcomes.

4. Reducing Structural and Financial Barriers

Widespread anxiety about treatment costs, especially among patients with low income or limited insurance coverage, underscores the need for policy action to reduce financial toxicity. Expanding coverage for immunosuppressants, laboratory testing, and outpatient follow-up under national health programs or social assistance schemes could significantly alleviate economic stress and its negative impact on mental well-being.

5. Developing a Multidisciplinary, Patient-Centered Model

Given the complex interplay between education, access, anxiety, and well-being, a multidisciplinary approach is essential. Post-KTx care should involve not only nephrologists and transplant surgeons but also psychologists, social workers, pharmacists, and patient navigators. Collaborative care models can ensure that patients receive comprehensive, continuous, and personalized support throughout the recovery journey.

### 4.5. Strengths and Limitations

This study offers several strengths that contribute valuable insights into the post-transplant experience of kidney recipients in Kazakhstan—a population that has received limited attention in the existing literature. First, it is one of the few studies in Central Asia to comprehensively assess the well-being of post-KTx patients using a validated tool (WHO-5 Well-Being Index) and to explore how healthcare accessibility, satisfaction with care, and psychological anxiety influence outcomes. Second, the inclusion of a relatively large and geographically diverse sample (*n* = 223) strengthens the generalizability of the findings across various urban and rural contexts within the country. Third, the use of multiple linear regression and mediation analyses allowed for a nuanced understanding of both direct and indirect effects of key factors, particularly in highlighting the mediating role of healthcare access and education in well-being.

Despite these strengths, this study has several limitations. Its cross-sectional design precludes any inference of causality; longitudinal studies are needed to determine how well-being evolves over time and how changes in access, anxiety, or care satisfaction influence outcomes. Self-reported data may also be subject to recall bias or social desirability bias, particularly in reporting satisfaction or psychological symptoms. In addition, the online and telephone-based recruitment methods may have excluded patients with limited internet access, reduced mobility, or lower health literacy, potentially underrepresenting the most vulnerable subgroups. While this study assessed psychological anxiety and satisfaction, it did not include clinical outcomes such as graft function, hospitalization, or medication adherence, which could further contextualize the findings. Additionally, while our study assessed post-transplant anxiety levels, it did not collect information on the use of psychiatric medications. This may represent an important unmeasured confounding or moderating factor, as pharmacological treatment could influence both anxiety levels and psychological well-being. Future research should include mental health treatment variables to clarify these relationships and better capture the complexity of psychological outcomes in post-KTx populations.

Future research should build on these findings using longitudinal designs, incorporate qualitative methods to capture in-depth patient perspectives, and explore interventions aimed at improving psychosocial well-being, particularly in underserved populations.

## 5. Conclusions

This study highlights the multifactorial nature of well-being among post-kidney transplant (KTx) patients in Kazakhstan, demonstrating that psychological health is shaped not only by individual characteristics but also by structural and contextual factors. Educational level and satisfaction with transplant-related information were positively associated with well-being, while difficulties in accessing healthcare and elevated post-transplant anxiety were significant negative predictors. Although geographic residence did not independently influence well-being, it exerted an important indirect effect through healthcare access barriers, especially among rural and small-town residents.

These findings underscore the need for a holistic and patient-centered approach to post-transplant care that prioritizes accessibility, mental health support, and health education. Addressing these modifiable determinants through targeted clinical strategies and inclusive health policies could significantly enhance the quality of life of transplant recipients and reduce disparities across regions. Ultimately, improving well-being in the post-KTx population requires not only medical management but also sustained investments in psychosocial and systemic support.

## Figures and Tables

**Table 1:** Study population (*n* = 223).

Variable	*n*	%
Gender		
Male	98	43.9
Female	125	56.1
Age group		
18–39 years	118	52.9
40–64 years	105	47.1
Residence		
Rural	33	14.8
Small-town	29	13.0
Regional-level city	75	33.6
National-level city	86	38.6
Educational level		
Middle school	11	4.9
High school	22	9.9
Special education (college)	58	26.0
Undergraduate	130	58.3
Postgraduate	2	0.9
Occupation		
Student	19	8.5
Employed	82	36.8
Self-employed	26	11.7
Unemployed	18	8.1
On disability benefits	69	30.9
Pensioner	9	4.0
Family status		
Single	66	29.6
Married	135	60.5
Divorced	17	7.6
Widow	5	2.2
Children		
No	79	35.4
Yes	144	64.6
Comorbidities		
No	83	37.2
Yes	140	62.8
KD ^1^ duration before KTx ^2^		
<1 year	46	20.6
1–3 years	74	33.2
4–5 years	22	9.9
>5 years	81	36.3

^1^ KD—kidney disease; ^2^ KTx—kidney transplantation.

**Table 2:** Satisfaction with post-KTx medical care (*n* = 223).

Satisfaction with	Very Dissatisfied	Dissatisfied	Partially Satisfied	Satisfied	Very Satisfied
The process of communication with medical staff	1 (0.4%)	11 (4.9%)	29 (13.0%)	69 (30.9%)	113 (50.7%)
The amount and quality of information you received about life after transplantation	3 (1.3%)	18 (8.1%)	36 (16.1%)	75 (33.6%)	91 (40.8%)
The level of post-KTx ^1^ care (e.g., condition monitoring, counselling, post-KTx check-ups)	6 (2.7%)	20 (9.0%)	44 (19.7%)	65 (29.1%)	88 (39.5%)
The quality of post-transplant medical care at the registered primary healthcare facility	16 (7.2%)	25 (11.2%)	53 (23.8%)	63 (28.3%)	66 (29.6%)

^1^ Kidney transplantation.

**Table 3:** Post-KTx ^1^ health and treatment-related anxiety (*n* = 223).

Items	Never	Rarely	Sometimes	Often	Always
Concern about the risk of infection due to taking immunosuppressants after transplantation	13 (5.8%)	28 (12.6%)	62 (27.8%)	30 (13.5%)	90 (40.4%)
Concern about how well the function of the transplanted kidney will be maintained	10 (4.5%)	23 (10.3%)	58 (26.0%)	29 (13.0%)	103 (46.2%)
Concern about poor laboratory test results during clinic visits	5 (2.2%)	29 (13.0%)	59 (26.5%)	37 (16.6%)	93 (41.7%)
Concern about how long health will remain stable after transplantation	13 (5.8%)	20 (9.0%)	54 (24.2%)	33 (14.8%)	103 (46.6%)
Concern about financial costs associated with prescribed post-transplant treatment	15 (6.7%)	19 (8.5%)	48 (21.5%)	37 (16.6%)	104 (46.6%)
Experience of depression or low mood after transplantation	40 (17.9%)	51 (22.9%)	61 (27.4%)	19 (8.5%)	52 (23.3%)
Concern about potential problems with the transplanted kidney and overall health due to taking immunosuppressive medications	23 (10.3%)	32 (14.3%)	57 (25.6%)	35 (15.7%)	76 (34.1%)

^1^ Kidney transplantation.

**Table 4:** Predictors of well-being among post-KTx patients (R^2^ = 0.242, F = 2.63, *p* < 0.001).

Predictor	Estimate	SE	t	Stand. Estimate	95% CI		*p*
					Lower CI	Upper CI	
Intercept	74.829	15.14	4.943				<0.001
Gender							
Female − Male	−0.934	3.29	−0.283	−0.038	−0.302	0.226	0.777
Age							
40–64 − 18–39 y.o.	1.837	3.96	0.463	0.075	−0.243	0.392	0.644
Residence							
Small town − Rural	0.306	6.09	0.050	0.012	−0.476	0.501	0.960
Regional-level city − Rural	1.510	5.12	0.295	0.061	−0.349	0.472	0.768
National-level city − Rural	−0.066	5.24	−0.013	−0.003	−0.423	0.418	0.990
Educational level	4.853	2.05	2.370	0.171	0.029	0.314	0.019
Occupation							
Pensioner − Disability	1.535	8.49	0.181	0.062	−0.618	0.714	0.857
Unemployed − Disability	−2.739	6.57	−0.417	−0.111	−0.638	0.415	0.677
Self-Employed − Disability	0.120	5.49	0.022	0.005	−0.436	0.445	0.983
Employed − Disability	−0.356	4.14	−0.086	−0.014	−0.346	0.317	0.932
Student − Disability	1.404	6.64	0.211	0.057	−0.475	0.590	0.833
Marital status							
Married − Single	−12.222	6.68	−1.830	−0.497	−1.032	0.038	0.069
Divorced − Single	−16.627	7.64	−2.177	−0.676	−1.228	−0.064	0.031
Widow − Single	−16.349	12.89	−1.268	−0.665	−1.698	0.369	0.206
Children (Yes − No)	7.259	6.37	1.139	0.295	−0.216	0.806	0.256
Comorbidities (Yes − No)	−5.902	3.41	−1.733	−0.240	−0.513	0.033	0.085
CKD duration before KTx	−1.300	1.38	−0.944	−0.062	−0.192	0.068	0.346
Difficulties with access to healthcare and medications	0.233	1.60	0.146	0.012	−0.146	0.170	0.884
Difficulties with availability of post-KTx follow-up	−4.102	1.65	−2.481	−0.216	0.387	−0.044	0.014
Satisfaction in communication with HCWs	0.230	3.01	0.076	0.008	−0.208	0.225	0.939
Satisfaction with information	7.003	2.85	2.461	0.287	0.057	0.517	0.015
Satisfaction with post-KTx care	−1.217	2.56	−0.475	−0.054	−0.278	0.170	0.635
Satisfaction with registered primary healthcare facilities	−3.545	1.88	−1.882	−0.176	−0.360	0.008	0.061
Post-KTx health and treatment-related anxiety	−3.772	1.66	−2.277	−0.154	−0.287	−0.021	0.024

**Table 5:** Indirect effect of residence on well-being through difficulties with access to healthcare and medications.

Type	Effect	Estimate	SE	*p*
Indirect	Residence ⟹ Difficulties with access to healthcare and medications ⟹ Well-being	1.877	0.613	0.002
Component	Residence ⟹ Difficulties with access to healthcare and medications	−0.354	0.079	<0.001
	Difficulties with access to healthcare and medications ⟹ Well-being	−5.302	1.269	<0.001
Direct	Residence ⟹ Well-being	1.055	1.559	0.499
Total	Residence ⟹ Well-being	2.932	1.554	0.059

**Table 6:** Indirect effect of educational level on well-being through difficulties with access to healthcare and medications.

Type	Effect	Estimate	SE	*p*
Indirect	Educational level ⟹ Difficulties with access to healthcare and medications ⟹ Well-being	1.186	0.569	0.037
Component	Educational level ⟹ Difficulties with access to healthcare and medications	−0.238	0.098	0.016
	Difficulties with access to healthcare and medications ⟹ Well-being	−4.979	1.209	<0.001
Direct	Educational level ⟹ Well-being	5.311	1.801	0.003
Total	Educational level ⟹ Well-being	6.498	1.849	<0.001

## Data Availability

The dataset supporting the conclusions of this article is available from the corresponding author on reasonable request.

## Abbreviations

The following abbreviations are used in this manuscript:

CKD	chronic kidney disease
ESRD	end-stage renal disease
HRQoL	health-related quality of life
KD	kidney disease
KTx	kidney transplantation
pmp	per million population
WHO	World Health Organization
